# Characterization of Post-Operative Hemodynamics Following the Norwood Procedure Using Population Data and Multi-Scale Modeling

**DOI:** 10.3389/fphys.2021.603040

**Published:** 2021-05-13

**Authors:** Jonathan Primeaux, Arash Salavitabar, Jimmy C. Lu, Ronald G. Grifka, C. Alberto Figueroa

**Affiliations:** ^1^Department of Biomedical Engineering, University of Michigan, Ann Arbor, MI, United States; ^2^C.S. Mott Children’s Hospital, University of Michigan Congenital Heart Center, Ann Arbor, MI, United States; ^3^Metro Heart and Vascular, Grand Rapids, MI, United States; ^4^Department of Surgery, University of Michigan, Ann Arbor, MI, United States

**Keywords:** hypoplastic left heart syndrome, computational fluid dynamics, Norwood procedure, systemic-to-pulmonary artery shunt, multi-scale modeling

## Abstract

Children with hypoplastic left heart syndrome (HLHS) must undergo multiple surgical stages to reconstruct the anatomy to a sustainable single ventricle system. Stage I palliation, or the Norwood procedure, provides circulation to both pulmonary and systemic vasculature. The aorta is reconstructed and attached to the right ventricle and a fraction of systemic flow is redirected to the pulmonary arteries (PAs) through a systemic-to-PA shunt. Despite abundant hemodynamic data available 4–5 months after Norwood palliation, data is very scarce immediately following stage I. This data is critical in determining post-operative success. In this work, we combined population data and computational fluid dynamics (CFD) to characterize hemodynamics immediately following stage I (post-stage I) and prior to stage II palliation (pre-stage II). A patient-specific model was constructed as a baseline geometry, which was then scaled to reflect population-based morphological data at both time-points. Population-based hemodynamic data was then used to calibrate each model to reproduce blood flow representative of HLHS patients. The post-stage I simulation produced a PA pressure of 22 mmHg and high-frequency oscillations within the flow field indicating highly disturbed hemodynamics. Despite PA mean pressure dropping to 14 mmHg, the pre-stage II model also produced high-frequency flow components and PA wall shear stress increases. These suboptimal conditions may be necessary to ensure adequate PA flow throughout the pre-stage II period, as the shunt becomes relatively smaller compared to the patient’s somatic growth. In the future, CFD can be used to optimize shunt design and minimize these suboptimal conditions.

## Introduction

**FIGURE 1:**
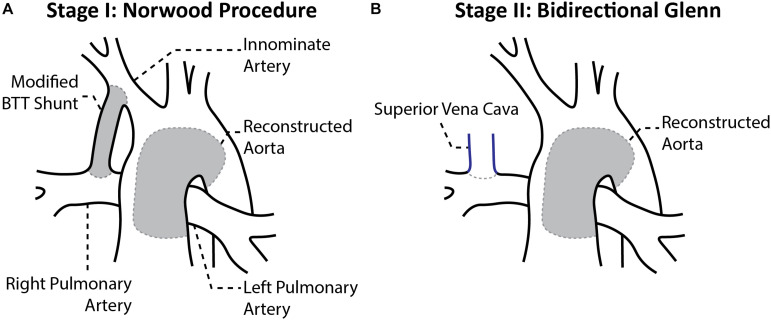
Anatomical configuration after the **(A)** stage I Norwood procedure and **(B)** stage II Bidirectional Glenn (Migliavacca et al., 2002).

Hypoplastic left heart syndrome (HLHS) is a congenital heart disease that, while affecting only 1 out of every 4,344 births, accounts for 40% of all neonatal cardiac deaths (Mai et al., 2010). HLHS is characterized by left ventricular inflow and aortic outflow tract hypoplasia that limits cardiac output and is rapidly fatal if untreated. Therefore, HLHS patients must undergo a series of three surgical procedures to palliate these complex anatomical defects into a single ventricle system. Stage I palliation, or the Norwood procedure, is commonly performed within the first week of life. The Norwood procedure consists of reconstructing the aortic arch and connecting it to the right ventricle, then placing a systemic-to-pulmonary artery (PA) shunt to enable blood flow to the PAs. Options for the shunt include a modified Blalock-Thomas-Taussig shunt (mBTTs) from the innominate artery to the PAs (Figure 1A), a central shunt from the ascending aorta to the PAs, or a Sano shunt from the right ventricle to the PAs. However, in this study it was decided to focus on the mBTTs approach as it is a common approach utilized for HLHS patients (Sano et al., 2004). Following stage I palliation, there are numerous reported complications, including reduced cardiac output, insufficient growth of the PAs, and stenosis at the shunt suture sites, all of which can affect mortality and hinder the success of the subsequent surgical procedures (Tweddell et al., 2002; Wells et al., 2005; Kawada, 2008). Despite surgical refinements of the Norwood procedure, the complications following stage I palliation lead to a mortality rate of 15–25% (Hornik et al., 2012). Stage I palliation is followed by two additional procedures: stage II, which consists of a superior cavopulmonary anastomosis [bidirectional Glenn (Figure 1B) or hemi-Fontan procedure] at 4–6 months of age, and stage III, the total cavopulmonary anastomosis (Fontan procedure) at 18–48 months of age). At stage II palliation, the mBTTs is removed, and superior vena cava flow is directed to the PAs (Figure 1B). Despite significant improvement over time, the highest risk for mortality is in the immediate post-stage I and in the interstage period between stage I and stage II (Mahle et al., 2000; Tweddell et al., 2002). While abundant hemodynamic data are obtained during pre-operative evaluation of pre-stage II (pre-S2) conditions, immediate post-stage I (post-S1) data are rarely reported. Despite many efforts to closely monitor the stage I post-operative course for HLHS patients, there remains a paucity of critically important hemodynamic data (specifically PA hemodynamic data). The consequence of this limited understanding of PA physiology is further magnified when considering PA stenosis occurs in nearly 50% of HLHS patients following stage I palliation (Griselli et al., 2006; Aiyagari et al., 2014). In this work, we seek to characterize the physiological mechanisms that can potentially contribute to understanding complications following stage I palliation. By combining clinical data and computational fluid dynamics (CFD) tools, we gain insight on hemodynamics immediately following stage I palliation and characterize the evolution of key hemodynamic indices between stage I and stage II.

Despite previous use of CFD models to study shunt placement, shunt designs, and various surgical configurations of HLHS patients, they often contain numerous shortcomings. These models have assumed purely 0D (lumped parameter models) or 3D rigid wall approaches and are often patient-specific models (therefore not reflecting population-based hemodynamic and anatomical characteristics) (Bove et al., 2003; Qian et al., 2010; Ceballos et al., 2012; Itatani et al., 2012; Moghadam et al., 2012; Baker et al., 2013; Arthurs et al., 2017; Fumero et al., 2017). Furthermore, these models are based solely on clinical data collected 4–5 months after initial palliation (pre-S2) leading to a limited understanding of the early post-operative conditions HLHS patients experience. Up to this point, there has not been a computational study that investigates the hemodynamics of HLHS patients immediately following stage 1 palliation. A computational fluid-structure interaction (FSI) model representing a broad patient population immediately post-S1 and through pre-S2, would lead to an enhanced understanding of the conditions and complications that HLHS patients experience. We seek to combine imaging and literature-based clinical data to develop computational models that are representative of HLHS patients for both post-S1 (within first week after palliation) and pre-S2 (within days before stage II palliation) conditions. These computational tools may allow us to gain detailed insights into the complex physiologic conditions these patients experience between stage I and stage II palliation (Arthurs et al., 2017).

## Materials and Methods

The development of both post-S1 and pre-S2 models required the careful compiling of clinical data and computational methodologies discussed herein. First, we will describe the general modeling approach that was taken to construct both models. Then, we will discuss the clinical data that was measured and collected to influence both models. Finally, we will outline the steps required to construct, calibrate, and parameterize both post-S1 and pre-S2 models.

### Overview

**FIGURE 2:**
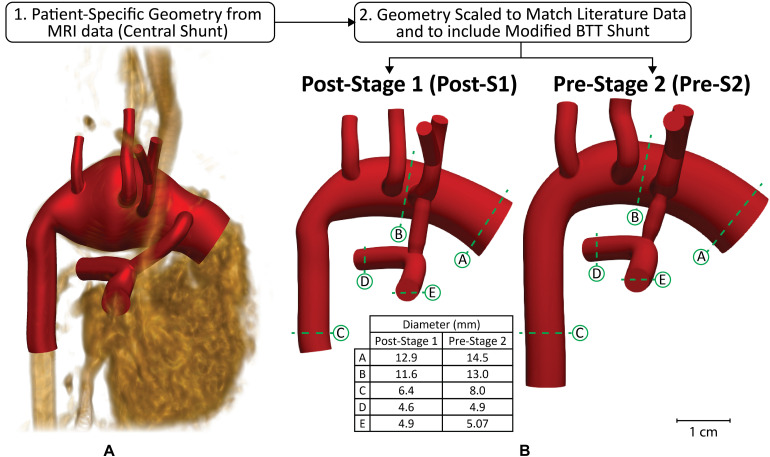
Modeling approach used to reconstruct anatomy of representative HLHS patient geometry at two time-points–post-S1 and pre-S2. Anatomy was constructed from **(A)** patient-specific MRI data that was then **(B)** scaled to match literature-based morphological data for both time-points (Machii and Becker, 1997; Mahle et al., 1998; Malec et al., 2003; Fiore et al., 2011; Bellsham-Revell et al., 2013; Aiyagari et al., 2014). The slight degree of tapering at each anastomosis of the shunt can be observed.

To construct geometries that represent a broad population of HLHS patients who have undergone the Norwood procedure, we began by constructing a patient-specific model as a representative baseline configuration (Figure 2A). Once this “template” model was constructed, morphological literature data was used to scale the geometry to reflect HLHS patient anatomy immediately following stage I palliation (post-S1) and prior to stage II palliation (pre-S2) (Figure 2B). Literature data reflecting post-S1 and pre-S2 hemodynamic conditions were applied to the respective models to simulate the complex hemodynamics these patients experience at both time-points. The outcome of this approach is a weighted-average (or “population-average”) model that represents a typical HLHS patient at the two time-points of interest–post-S1 and pre-S2. The study was approved by the University of Michigan Board of Review.

### Clinical Data

#### Patient-Specific Data

Clinical data of a 3-week old HLHS patient was acquired. At birth, this patient exhibited anatomical characteristics typical for HLHS patients (mitral and aortic atresia, non-restrictive atrial septal defect) and underwent the Norwood procedure at 4 days of age (Jonas et al., 1994). During stage I palliation, the surgeon found the patient’s innominate artery suboptimal for shunt placement, so the decision was made to connect the systemic-to-pulmonary shunt from the ascending aorta to the main pulmonary artery (MPA) (see Figure 2A). Two weeks after stage I palliation, magnetic resonance angiography (MRA) data were acquired to assess post-operative characteristics including PA stenosis, palliation of the aortic arch, and possible shunt occlusion. Furthermore, phase-contrast magnetic resonance imaging (PC-MRI) data were collected at the ascending aorta, transverse aortic arch, descending aorta, and bilateral PAs to assess critical parameters such as cardiac output, pulmonary-to-systemic flow ratio (Qp:Qs), and valve regurgitation. Blood pressure measurements were collected using a brachial cuff concurrent with the MRA and PC-MRI examinations.

At the time of imaging data acquisition, the patient continued to exhibit anatomical characteristics of a typical HLHS patient with no additional compromising anomalies (i.e., aortopulmonary collateralization, PA stenosis, shunt occlusion). Of note, the distal transverse aortic arch of the patient was markedly dilated (Figure 2A). The proximal right PA was also mildly angulated, a common finding in patients after stage I palliation (Mahle et al., 1998).

#### Literature Population Data

In order to develop representative computational models of typical HLHS patients, the acquired patient-specific clinical data was carefully combined with literature data on anatomy (Figure 2) and hemodynamics (Table 1) for the two time-points of interest: post-S1 and pre-S2 (Rychik et al., 2002; Maher et al., 2003; Mahle et al., 2003; Mair et al., 2003; Malec et al., 2003; Pizarro et al., 2003; Ballweg et al., 2005; Cua et al., 2006; Ghanayem et al., 2010; Fiore et al., 2011; Bellsham-Revell et al., 2013; Aiyagari et al., 2014). All literature values were based on patients born with HLHS who had undergone the Norwood procedure with a surgically placed mBTTs.

**TABLE 1:** Literature-based weighted means of hemodynamic parameters for post-S1 and pre-S2.

**Parameter**	**Post-Stage I**	**Pre-Stage II**
Age (months)	0.25	5.09
Body Surface Area (m^2^)	0.21	0.31
Arterial Pressure (mmHg)		
Systolic Pressure	72.4	100.3
Diastolic Pressure	37.8	40.98
Mean Pressure	50.3	60.5
Pulse Pressure	34.4	59.3
Pulmonary Artery Mean Pressure (mmHg)	*	14.2
Cardiac Output (L/min)	1.15^†^	1.7
Pulmonary-Systemic Flow Ratio (Q_*p*_:Q_*s*_)	1.7	1.28

*Indicates data not available. ^†^Indicates allometric scaling. (Rychik et al., 2002; Maher et al., 2003; Mahle et al., 2003; Mair et al., 2003; Malec et al., 2003; Pizarro et al., 2003; Ballweg et al., 2005; Cua et al., 2006; Ghanayem et al., 2010; Fiore et al., 2011; Bellsham-Revell et al., 2013; Aiyagari et al., 2014).

To obtain literature-based values, a detailed review of all available literature that included hemodynamic and morphological data of HLHS patients was conducted. Inclusion criteria included: (1) patient populations presented with HLHS, (2) the use of the mBTTs during the Norwood procedure, (3) size of surgically placed shunt either 3.5 mm or 4.0 mm, and (4) reporting morphological and/or hemodynamic data within the first week post-S1 and/or at pre-S2 follow-up. Exclusion criteria included: (1) study population overlapped with another study and (2) patient population presented with coexisting cardiac anomalies. The search strategy yielded 15 included studies (Tables 2, 3).

**TABLE 2:** Hemodynamic and morphological literature data for healthy patients (left) and HLHS post-S1 patients.

**Parameters**	**References**									
	**Healthy Patients**		**Hypoplastic Left Heart Syndrome Patients**							
	**Machii and Becker, 1997**	**Pettersen et al., 2008**	**Mahle et al., 1998**	**Malec et al., 2003**	**Mahle et al., 2003**	**Mair et al., 2003**	**Rychik et al., 2002**	**Cua et al., 2006**	**Ghanayem et al., 2010**	**Pizarro and Norwood, 2003**
*n*	7	813	50	31	22	18	24	37	117	20
Age (days)	5	–	19.9 (2–46)	13.9 ± 8.4	5	10.44	–	4 (2–17)	7.2 ± 6.5	4.5 ± 3.6
Arterial Systolic Pressure (mmHg)	–	–	–	77	78.2 ± 8.4	62.8 (57.5–72)	78 ± 11	64 ± 7	–	76.5
Arterial Diastolic Pressure (mmHg)	–	–	–	39	38.4 ± 4.4	37.3 (32–41)	37 ± 7	36.5	–	39
Mean Arterial Pressure (mmHg)	–	–	–	–	–	–	–	47	51 ± 6	52.3
Qp:Qs	–	–	–	–	–	–	1.7 ± 1	–	1.7 ± 0.9	–
Body Surface Area (mm^2)	–	–	0.23 ± 0.02	–	–	–	–	–	–	–
LPA Diameter (mm)	–	4.6	–	–	–	–	–	–	–	–
RPA Diameter (mm)	–	4.9	–	–	–	–	–	–	–	–
Transverse Arch Diameter (mm)	–	–	11.6 ± 0.22	–	–	–	–	–	–	–
Descending Aorta Diameter (mm)	6.7 ± 0.8	6.1	–	–	–	–	–	–	–	–

*All data collected from HLHS patient were obtained within 48 h following stage I palliation. Values represent means ± standard deviations or medians with ranges.*

**TABLE 3:** Hemodynamic and morphological literature data for patient pre-S2.

	**References**							
**Parameters**	**Aiyagari et al., 2014**	**Bellsham-Revell et al., 2013**	**Ballweg et al., 2005**	**Malec et al., 2003**	**Mahle et al., 2003**	**Mair et al., 2003**	**Maher et al., 2003**	**Fiore et al., 2011**
Number of Patients (*n*)	170	58	78	31	22	10	10	19
Age at Cath (months)	4.5 ± 1.5	5.28 ± 2.16	6.23 (2.67–16.3)	6.04 ± 1.0	4.8	3	5.2	5 ± 1.0
Aortic systolic pressure (mmHg)	–	–	91 (68–135)	108 ± 14.6	–	–	105.8 ± 14.9	103 ± 14
Aortic diastolic pressure (mmHg)	35 ± 7	–	40 (20–57)	51.6 ± 9.5	–	–	41.5 ± 10.9	46 ± 14
Mean PA pressure (mmHg)	14 (13–17)	–	15 (8–22)	–	11 (8–16)	14 (14–17)	16.6 ± 3.74	–
Qp:Qs	1.1 (0.8–1.5)	–	1.6 (0.47–4.6)	1.41 ± 0.7	0.9 (0.4–1.6)	1.6 (1.2–1.6)	1.42 ± 0.36	–
Cardiac Index (L/min/m^∧^2)	–	5.5 ± 2.0	–	–	–	–	–	–
Body Surface Area (mm^∧^2)	0.31 ± 0.04	–	0.31 ± 0.04	–	–	–	–	–
LPA Diameter (mm)	4.8 (4.0–6.0)	6.01 ± 5.83	5.8 (2.7–11.8)	–	–	–	–	7.6 ± 3.7
RPA Diameter (mm)	5 (4.0–6.1)	8.1 ± 5.5	6.2 (2.7–12.3)	–	–	–	–	6.3 ± 4.5
Ascending Aorta Diameter (mm)	–	14.51 ± 7.82	–	–	–	–	–	–
Transverse Arch Diameter (mm)	–	12.96 ± 7.7	–	–	–	–	–	–
Descending Aorta Diameter (mm)	–	8.04 ± 5.1	–	–	–	–	–	–

*All data were collected from HLHS undergoing pre-S2 clinical follow-up. Values represent means ± standard deviations or medians with ranges.*

Following the Norwood procedure, post-S1 data was collected during the post-operative hospitalization (mean age of 7 days) (Table 2). Hemodynamic data were obtained from brachial cuff pressure, cardiac catheterization, Doppler echocardiography, and oxygen saturation of blood samples (Rychik et al., 2002; Mahle et al., 2003; Mair et al., 2003; Malec et al., 2003; Pizarro and Norwood, 2003; Cua et al., 2006; Ghanayem et al., 2010). Reported hemodynamic data included systemic arterial pressure and Q_*p*_:Q_*s*_ (Table 2). Morphological data was obtained using echocardiography (Machii and Becker, 1997; Mahle et al., 1998; Pettersen et al., 2008). Regions of native tissue (supra-aortic arteries, descending aorta, LPA, and RPA) were assumed to exhibit normal morphological characteristics of children less than 1-month of age (Machii and Becker, 1997; Pettersen et al., 2008). This assumption cannot be applied to the reconstructed region of the aorta. Measurements of the reconstructed arch in HLHS patients during the post-operative period following the Norwood procedure were applied from the ascending aorta to the transverse arch (Mahle et al., 1998).

Pre-S2 data was collected during pre-operative follow-up for stage II palliation (mean age of 5.1 months). Hemodynamic data was obtained from cardiac catheterization and PC-MRI (Maher et al., 2003; Mahle et al., 2003; Mair et al., 2003; Malec et al., 2003; Ballweg et al., 2005; Fiore et al., 2011; Bellsham-Revell et al., 2013; Aiyagari et al., 2014). Reported hemodynamic data included systemic arterial pressure, PA mean pressure, cardiac output, and Qp:Qs (Table 3). Morphological data was obtained using MRA and echocardiography (Machii and Becker, 1997; Ballweg et al., 2005; Fiore et al., 2011; Bellsham-Revell et al., 2013; Aiyagari et al., 2014). The descending aorta and supra-aortic arteries were assumed to exhibit normal morphological characteristics of children from 1-month to less than 1 year in age (Machii and Becker, 1997). Following the Norwood procedure, the PAs are known to experience unfavorable hemodynamic conditions leading to altered growth, so pre-S2 measurements of the LPA and RPA were applied (Ballweg et al., 2005; Bellsham-Revell et al., 2013; Aiyagari et al., 2014). Measurements of the reconstructed aortic arch in HLHS patients during pre-S2 assessment were applied from the ascending aorta to the transverse arch (Bellsham-Revell et al., 2013).

#### Statistical Analysis

Weighted mean values of collected literature-based hemodynamic and morphological parameters were calculated using the fixed effect model (Table 1; Borenstein et al., 2010). By considering sample size and standard deviations of each study, the fixed effect model applies more weight to studies that report more precise data, therefore providing data more representatives of typical HLHS patients for the two time-points studied. Some studies within the literature review did not report the standard deviation of measured parameters. In this case, a method commonly applied to approximate standard deviation (or variance) from reported median, range, and sample size was used (Hozo et al., 2005). All literature-based weighted means of hemodynamics for both post-S1 and pre-S2 can be found in Table 1.

### CFD Simulations

Computational modeling tools were applied to calculate the hemodynamic conditions of HLHS patients following the Norwood procedure. CFD is a well-established methodology that enables the calculation of the velocity and pressure fields of an incompressible fluid by solving the Navier–Stokes equations. In this study, to perform FSI simulations we must define: (1) geometric representation of the regions (blood vessels) of interest, (2) inflow and outflow boundary conditions representing pressure and velocity of typical HLHS patients, and (3) material properties for all vessel walls.

#### Geometric Modeling and Mesh Generation

Representative FSI models of post-S1 and pre-S2 HLHS patients were constructed using the cardiovascular modeling and blood flow simulation software package CRIMSON^1^ (Arthurs et al., 2020). A patient-specific model was first reconstructed from the MRA data (Figure 2A). This model included the ascending and descending aorta, left and right PA, left and right common carotid arteries, left and right subclavian arteries, and the surgically placed systemic-to-PA shunt. After reconstructing the patient-specific anatomy, the geometry was scaled to reflect literature-based morphological data measured post-S1 and during the standard clinical follow-up pre-S2 (Figure 2B; Machii and Becker, 1997; Mahle et al., 1998; Malec et al., 2003; Fiore et al., 2011; Bellsham-Revell et al., 2013; Aiyagari et al., 2014). Furthermore, the central shunt of the patient-specific geometry was replaced with a mBTTs shunt as this configuration is utilized more frequently in current practice. When making a significant change to the geometry, such as this, it is key to remain consistent with all remaining steps of the modeling process to avoid any misleading simulation results. Therefore, all data used during scaling and calibration were collected from patients with the specific mBTTs surgical configuration. Of note, the PA and descending aorta of the post-S1 model reflects diameters of healthy subjects as these regions of tissue are not reconstructed during the Norwood procedure and have not undergone significant remodeling as a result of palliation. The mBTTs was modeled with a diameter of 3.5 mm as this was the most common shunt size reported in the literature data. Furthermore, a small degree of tapering was modeled at each anastomosis of the shunt. “Pinching” at the shunt suture line has been reported to immediately impose a 10% diameter reduction at each anastomosis (Dobrin et al., 1998; Figure 2). This diameter reduction was applied to the post-S1 model. During calibration of the pre-S2 model, it was found that a 17% diameter reduction (bringing the shunt diameter to 2.9 mm) was required to reproduce PA hemodynamics found in the literature. This additional degree of pinching in the pre-S2 model could be attributed to intimal hyperplasia commonly occurring at suture sites in mBTTs patients, and is in line with previously reported values (Gladman et al., 1997; Wells et al., 2005).

Isotropic mesh generation using linear tetrahedral elements resulted in 505,964 elements for the post-S1 model and 743,456 elements for the pre-S2 model. Preliminary simulations were performed with these meshes and gradient-based mesh adaptive techniques were utilized to refine the finite element mesh in regions of high velocity gradients (Sahni et al., 2006). This produced adapted meshes consisting of 1,167,047 and 1,623,130 elements for the post-S1 and pre-S2 models, respectively. The results reported in this study used these finer meshes.

#### Boundary Condition Specification

**FIGURE 3:**
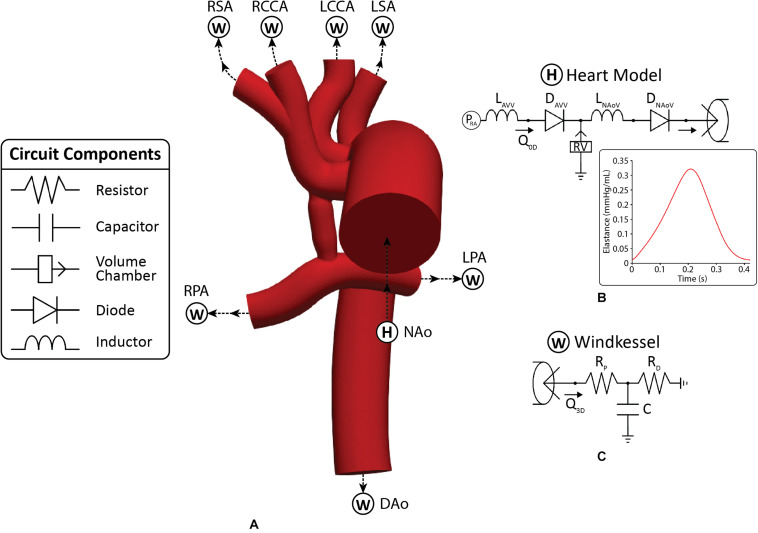
**(A)** The 3D-0D multi-domain framework utilized to simulate hemodynamics in both post-S1 and pre-S2 models. The geometry corresponding to the pre-S2 model is shown. Outlets with “W” correspond to a Windkessel boundary condition **(B)**. Outlet with “H” corresponds to the heart model boundary condition. **(B)** 0D heart model coupled to the ascending aorta inlet face. A plot of time-varying elastance to model the contraction and relaxation of the right ventricle (RV) is included. **(C)** Three-element Windkessel model representing the distal vasculature applied to all outlets. Abbreviations: L, inductor; D, diode; RPA, right pulmonary artery; LPA, left pulmonary artery; P, pressure; Q, flow; RV, right ventricle; RA, right atrium; R_*p*_, proximal resistance; R_*d*_, distal resistance; C, compliance; AVV, atrioventricular valve; NAoV, neo-aortic valve. Calibrated LPN parameter values are listed in Table 4.

Each model consisted of one inlet (ascending aorta) and seven outlets (all other modeled vessels). A multi-domain modeling approach was utilized to characterize blood flow. This method couples the non-linear incompressible three-dimensional Navier–Stokes equations describing velocities and pressures in 3D reconstructed vessels with a series of 0D lumped parameter networks (LPN) that capture distal hemodynamic behavior (resistance and compliance) as well as ventricular function (Figure 3). This approach has been successfully implemented to study single ventricle physiology (Moghadam et al., 2012; Arthurs et al., 2017). The CRIMSON Netlist Editor Boundary Condition Toolbox enables the introduction of dynamic, customizable three-element Windkessel models at the outlets and a heart model at the inlet (Figure 3A), and even coupling disconnected 3D domains (e.g., separate systemic and pulmonary geometries) in a closed-loop manner (Silva Vieira et al., 2018). Three-element Windkessel models were applied to each outlet to represent the resistance and compliance of the distal vasculature (Figure 3C; Vignon-Clementel et al., 2010). A custom 0D lumped parameter heart model was applied at the inflow of the neo-aorta (Figure 3B; Lau and Figueroa, 2015). The heart model included a series of 0D components that represented the right atrial and neo-aortic valves and the contraction of the right ventricle (Figure 3B). The contraction and relaxation of the right ventricle was simulated using time-varying elastance function (Figure 3B). The valves were modeled as diodes and only permitted forward flow.

**TABLE 4:** Tuned component parameters for the Windkessel models at the outlets of both the post-S1 and pre-S2 models.

	***R*_*p*_**	***R*_*d*_**	***C***
**Post-Stage I**			
LPA	0.053	0.466	0.134
RPA	0.039	0.402	0.250
DAo	0.100	1.718	1.326
RC	0.419	7.455	1.516
RS	0.498	7.376	1.341
LC	0.298	5.293	1.524
LS	0.397	7.057	1.535
**Pre-Stage II**			
LPA	0.058	0.192	0.239
RPA	0.041	0.163	0.444
DAo	0.170	1.175	2.357
RC	0.667	4.823	0.750
RS	0.781	4.709	0.645
LC	0.473	3.424	0.755
LS	0.631	4.566	0.761

**FIGURE 4:**
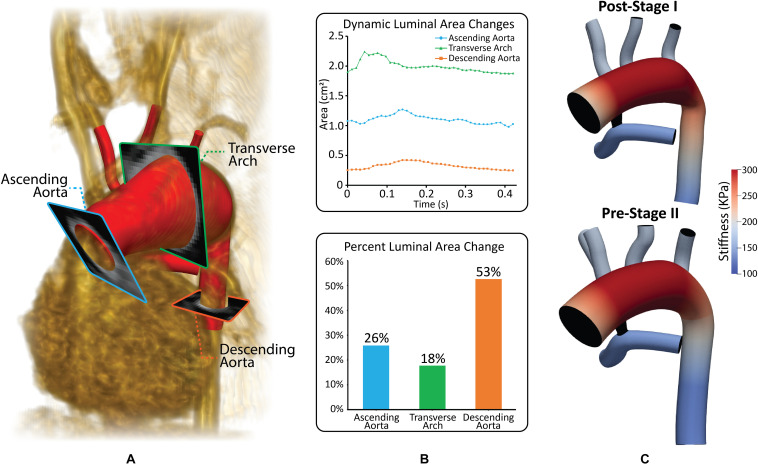
**(A)** Reconstructed geometry with volume-rendering of MRA data and three different PC-MRI planes. **(B)** Plot of dynamic luminal area changes and percent luminal area changes at the ascending aorta, transverse arch and descending aorta PC-MRI planes. **(C)** Material properties applied to both the post-S1 and pre-S2 models. Stiffness values were calculated based on patient-specific pressure measurements and dynamic luminal area changes measured from MRI data (Hirai et al., 1989).

*The units of resistance *R*_*p*_ and *R*_*d*_ are Pa⋅s/mm^3^. The units of compliance C are mm^3^/Pa. Abbreviations: LPA, left pulmonary artery; RPA, right pulmonary artery; DAo, descending aorta; RC, right common carotid; RS, right subclavian; LC, left common carotid, LS, left subclavian. See Figure 4 for the referenced boundaries.*

#### Fluid-Structure Interaction

Most CFD studies of HLHS patient hemodynamics have assumed rigid walls for all vessels. While this assumption can produce a good estimate of the velocity field, it has a significant impact on the pressure field and estimates of the LPN parameter values that could lead to misleading results. In this work, the “Coupled Momentum” FSI method was used (Figueroa et al., 2006). This required material properties, specifically linearized stiffness and thickness to be specified for each vessel. A uniform vessel thickness of 1.5 mm was applied to the aorta and PAs for both models, and a spatially varying thickness of 15% of the local vessel radius was specified for the aortic upper branches (van Meurs-van Woezik et al., 1983; Roccabianca et al., 2014). Linearized stiffness was determined using the patient-specific MRA data on dynamic luminal area (systolic to diastolic range), and pressure cuff data (section “Patient-Specific Data”), see Figure 4B (Hirai et al., 1989). Stiffness values were kept constant for both models, given that vessel wall properties do not significantly change within the first year of life (Figure 4C; Senzaki et al., 2002). The material properties determined are in line with reported values for HLHS patients following the Norwood procedure (Cardis et al., 2006). The reconstructed region of the aorta (transverse arch) exhibits the highest values of stiffness as it primarily consists of synthetic graft material. The ascending and descending aorta and PAs have lower stiffness since they consist of native tissue, and, as a result, are more distensible (Figure 4C). Finally, the shunt was assumed to be rigid.

#### Boundary Condition Parameterization

The parameterization of the multi-domain model seen in Figure 4 consisted of two main steps. In Step 1, a flow waveform was prescribed at the aortic inflow, and a calibration of the outflow branch’s LPN parameters was performed. In Step 2, upon calibration of outflow LPN parameters, a custom heart model was prescribed at the aortic inflow and calibrated (Xiao et al., 2013).

##### Step 1

The measured PC-MRI aortic inflow waveform was scaled to match the cardiac output found in literature for both the post-S1 and pre-S2 models. Calibration of LPN parameters was iteratively performed by running simulations on 60 cores in the University of Michigan high-performance computing cluster ConFlux. Simulations were run until cycle-to-cycle periodicity was reached in the pressure field, which typically took five cardiac cycles. Each simulation took approximately 48 h to complete depending on mesh size. Initial parameters of Windkessel elements (resistance and compliance) of all outlets were calculated using the iterative method described by Xiao et al. (2013). Then, total resistances of the Windkessel elements for both models were calibrated until the mean pressures and mean flow at each outlet matched within 10% of the data found in literature. Then, the Windkessel compliances were calibrated until the desired pulse pressure was achieved within 10%.

##### Step 2

The prescribed inflow was replaced by a custom 0D lumped parameter heart model at the inflow of the neo-aorta. The right atrial pressure (P_*RA*_) and parameters of the elastance function were adjusted until cardiac output was matched within 5% of values found in literature (Arthurs et al., 2017). Once the cardiac output was matched, small adjustments were made to the outflow boundary conditions until all hemodynamic parameters were matched within 10% of the data found in literature. The final numerical values for the components of the LPN outflow Windkessel and the heart model are presented in Tables 4, 5, respectively. The resultant Windeasel parameters for the pre-S2 case compare well to previously published values (Arthurs et al., 2017). Unfortunately, there are no previously published values for the post-S1 case to compare resultant Windkessel parameters.

**TABLE 5:** Tuned component parameters for the heart models at the inlets of both the post-S1 and pre-S2 models.

**Parameter**	**Value**
**Post-Stage 1 (Post-S1)**	
RAVV open resistance	3.3 × 10^–3^
RAVV inertance	6.667 × 10^–5^
Neo-aortic valve open resistance	1.0 × 10^–3^
Neo-aortic valve inertance	1.0 × 10^–5^
**Pre-Stage 2 (Pre-S2)**	
Right Atrial Pressure	5.4
RAVV open resistance	3.3 × 10^–3^
RAVV inertance	6.667 × 10^–5^
Neo-aortic valve open resistance	1.0 × 10^–3^
Neo-aortic valve inertance	1.0 × 10^–5^
Right Atrial Pressure	7.2

*The units of resistance are Pa⋅s/mm3. The units of inductance are Pa⋅s2/mm3. The units of pressure are mmHg. Abbreviations: RAVV, Right Atrioventricular Valve.*

## Results

### Calibration Against Hemodynamic Data

**FIGURE 5:**
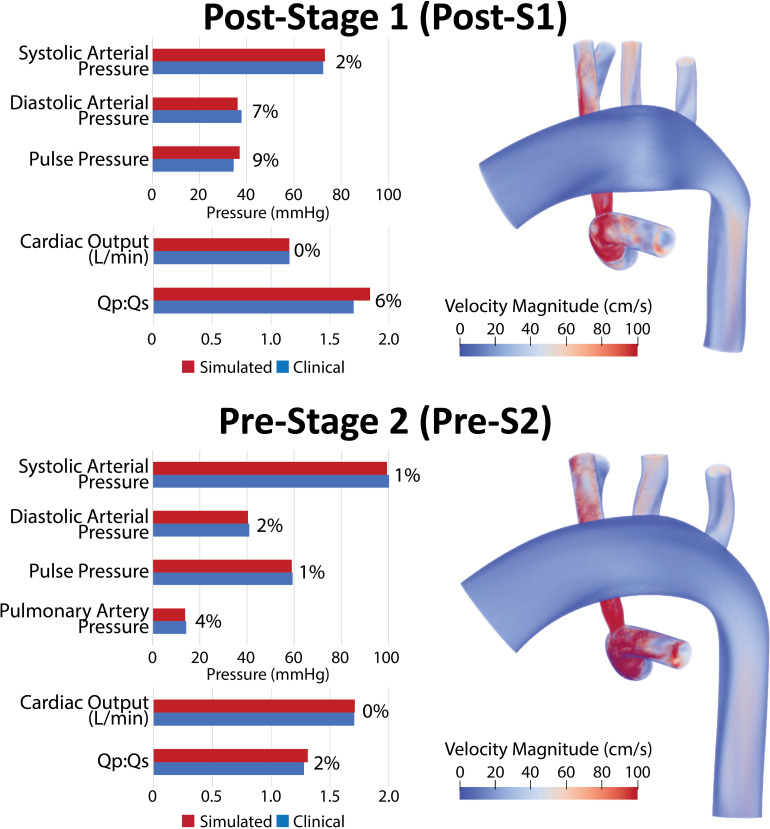
Left: Comparison of simulated results (red) and literature-based clinical data (blue) of hemodynamic indices for post-S1 and pre-S2 models. All indices were matched within 10% of literature values. Right: Volume renders of FSI simulations velocity maps.

Model calibration was initially attempted assuming rigid walls; however, we were uncapable unable of matching mean pressure and flow data while simultaneously matching pulse pressure. Once utilizing the more physiologically relevant approach of deformable walls, we were able to match all available population-based hemodynamic literature data. Both post-S1 and pre-S2 models were successfully calibrated to closely match literature-based hemodynamics on systemic pressure, cardiac outflow, Qp:Qs and, for the pre-S2 model, PA mean pressure. Simulated hemodynamic indices were within 8% of the literature-based data (Table 1) for both models (Figure 5).

### Simulated Flow and Pressure Waveforms

**FIGURE 6:**
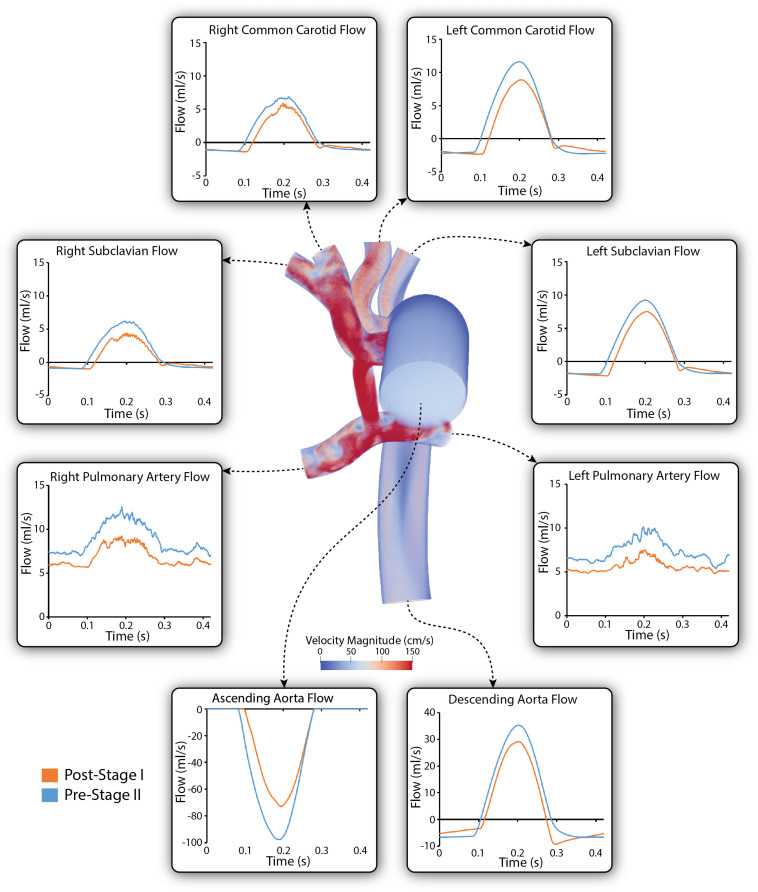
Simulated pulsatile flow waveforms at all boundaries of the 3D domain for both post-S1 (orange) and pre-S2 (blue) models. All outflows are plotted as positive and all inflows are plotted as negative. Note that three different *y*-axis scales have been used: one for the descending aorta, another for the ascending aorta and a third for the remainder of the outlets. A volume rendering of velocity magnitude for the pre-S2 model is shown during peak-systole (*t* = 0.21 s).

Figure 6 shows a comparison of the simulated pulsatile flow waveforms at all outlets for both the post-S1 and pre-S2 models. The mean flow through all outlets increased from post-S1 to pre-S2. This is to be expected as, in agreement with literature data, there was a 38% increase in cardiac output between stage I and stage II (Table 1). Despite Qp:Qs decreasing from post-S1 (1.8) to pre-S2 (1.3), the mean PA flow increased from post-S1 (0.75 L/min) to pre-S2 (0.97 L/min). The mean flow observed at the right common carotid and subclavian arteries is lower compared to that observed at the left common carotid and subclavian arteries. The waveforms within the right common carotid and right subclavian arteries present high-frequency disturbances in peak systole, which are absent on their left side counterparts. This can be attributed to the “run-off” of flow through the mBTTs to the pulmonary circulation that is commonly seen within HLHS patients (Kawada, 2008). Diastolic backflow is observed in the descending aorta and the supra-aortic vessels for both models. The overall change in the shape of the flow waveforms from post-S1 to pre-S2 is subtle.

**FIGURE 7:**
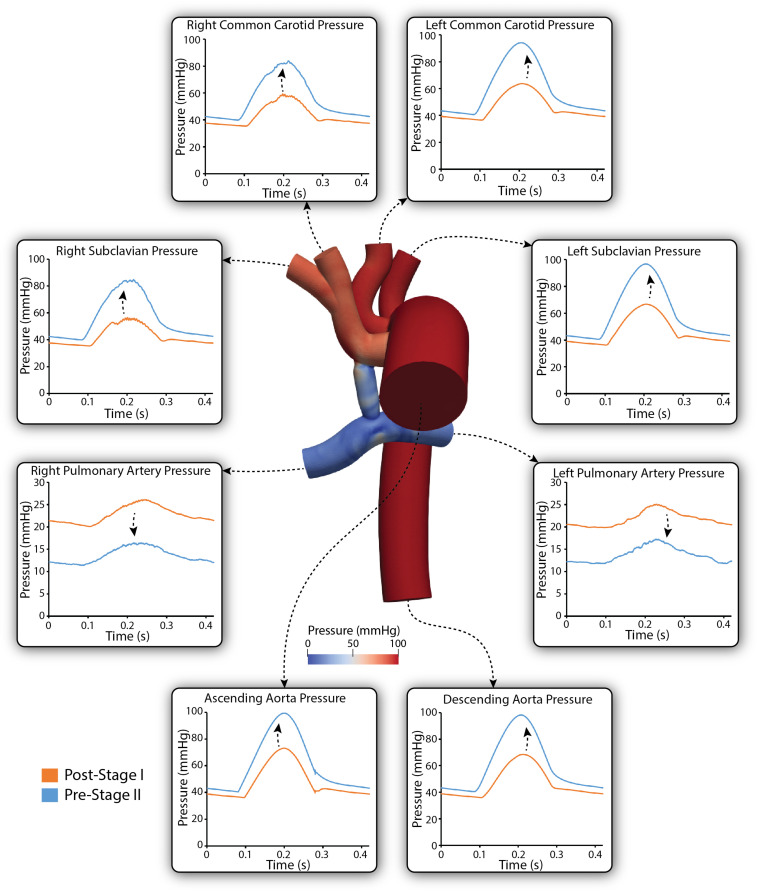
Simulated pulsatile pressures at all boundaries of the 3D domain for both post-S1 (orange) and pre-S2 (blue) models. Arrows indicate whether pressure increased or decreased from post-S1 to pre-S2. Note that two different *y*-axis scales have been used: one for the LPA and RPA, and a second for the remainder of the outlets. A pressure coloring on the surface of the pre-S2 model is shown during peak-systole (*t* = 0.21 s).

Figure 7 shows a comparison of the simulated pulsatile pressure waveforms at all outlets for both the post-S1 and pre-S2 models. While the simulated mean systemic arterial pressure increased from post-S1 (47.5 mmHg) to pre-S2 (60 mmHg), the PA mean pressure decreased substantially from approximately 22 to 14 mmHg. Lower mean pressures and subtle high-frequency oscillations in peak systole were observed at the right common carotid and subclavian arteries compared to the left common carotid and subclavian arteries. This is similar to the “run-off” phenomenon observed in mean flow received by the right common carotid and subclavian arteries (Kelleher et al., 2006).

### Disturbed Pulmonary Artery Hemodynamics

**FIGURE 8:**
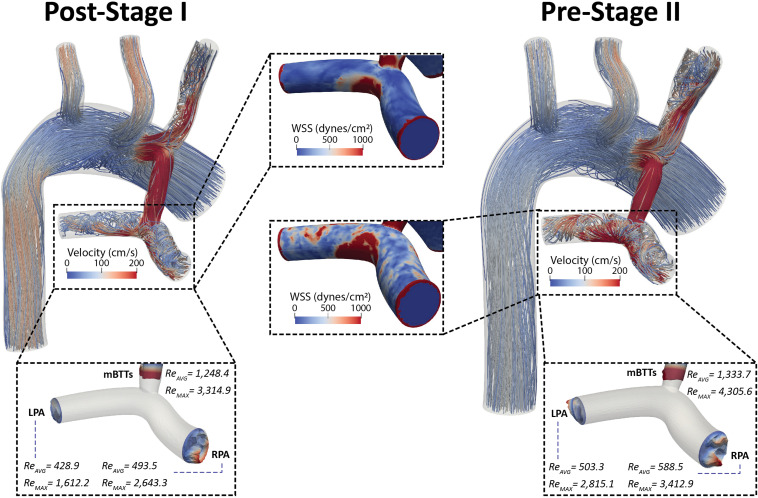
Velocity streamlines for both post-S1 and pre-S2 models during peak systole (*t* = 0.21 s). Disturbed flow patterns are prominent at the mBTTs-PA anastomosis and propagate throughout the PAs. Surface plots of WSS (center) in the PAs are presented for both models. There are concentrations of high WSS where the mBTTs flow impacts the wall of the PA. The WSS experienced in the PAs slightly increases during the interim period between stage I and II. Finally, velocity profiles at the mBTTs, LPA, and RPA (bottom) and their corresponding time average and peak systolic Reynolds numbers. High Reynolds numbers (>2,000) are indicative of turbulence and can be seen within the mBTTs and PAs. These Reynolds numbers increase from post-S1 to pre-S2.

High-frequency oscillations in pulmonary flow, indicative of hemodynamic disturbances (Tossas-Betancourt et al., 2020), were observed in both post-S1 and pre-S2 models. Disturbed PA flow is further illustrated in Figure 8. As seen in the velocity streamlines, the parallel velocity streamlines within the shunt become highly disturbed in the region where the shunt meets the main PA, propagating these disturbances throughout the PAs.

Wall shear stress (WSS) is another key regulator of vascular biology. It is known that spatially disturbed WSS may lead to pathological remodeling (Dolan et al., 2013). At post-S1 the PAs experience high values of WSS and continue to increase throughout the pre-S2 period.

Disturbed PA hemodynamics are further supported by large Reynolds numbers within the shunt and left and right PAs (Figure 8), indicating transitional/turbulent flow regimes. We calculated time average (Re_*avg*_) and peak systolic (Re_*max*_) Reynolds numbers at the shunt and each of the PA outlets. Peak systolic Reynolds numbers within the mBTTs (3,314.9 and 4,305.6 for post-S1 and pre-S2, respectively) were indicative of turbulence. It is also noteworthy that all calculated Reynolds numbers increase from post-S1 to pre-S2 indicating the hemodynamics become more disturbed throughout the pre-S2 period. In this paper, we adopted a “Direct Numerical Simulation” approach, and avoided the use of turbulence models. Convergence of the reported Reynolds number estimates would require much more refined finite element meshes however and is therefore outside the scope of this work.

### Mesh Independence and Numerical Accuracy

**FIGURE 9:**
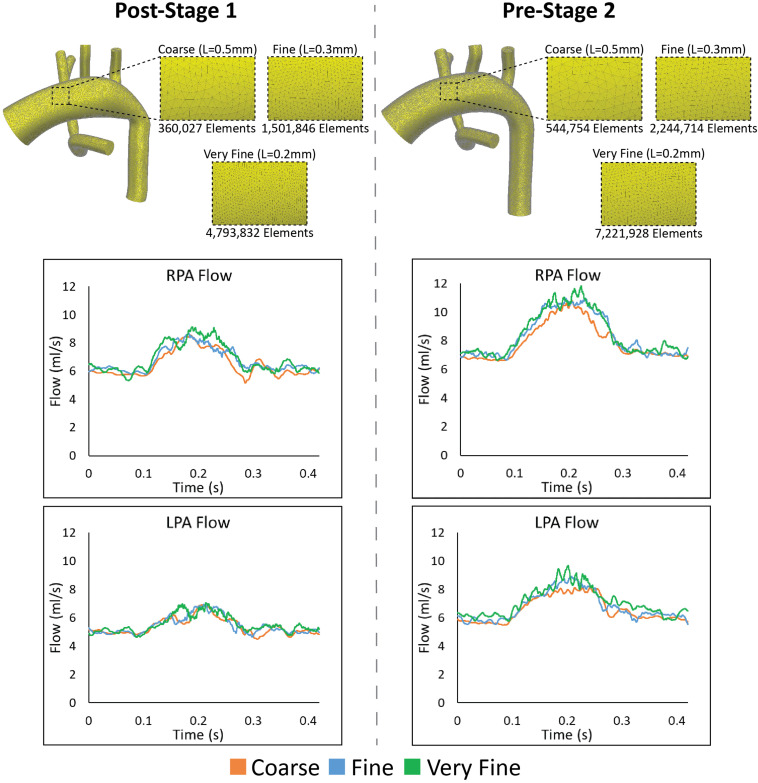
Top: The various isotropic linear finite element meshes generated for the mesh-independence analysis for both Post-S1 and Pre-S2 models. Finer meshes are generated by incrementally decreasing the global element size (L). Bottom: Results from the mesh independence analysis. Flow at the LPA and RPA are plotted for both Post-S1 and Pre-S2 at each mesh size–coarse (orange), fine (blue), and very fine (green). As seen in each plot, there is little alteration in mean flow values in the PAs and the high-frequency oscillation is present in all cases.

To ensure that the disturbed hemodynamics observed in the PAs were not a result of numerical error, we performed a mesh independence analysis. Three increasingly fine isotropic linear tetrahedral element meshes were generated for both Post-S1 and Pre-S2 models. Mesh resolution was increased by incrementally decreasing the global element size (*L*) for each mesh generated–coarse (*L* = 0.5 mm), fine (*L* = 0.3 mm), and very fine (*L* = 0.2 mm). The coarse, fine, and very fine meshes of the Post-S1 model consisted of 360,027 elements, 1,501,846 elements, and 4,793,832 elements, respectively (Figure 9). The coarse, fine, and very fine meshes of the Pre-S2 model consisted of 544,754 elements, 2,244,714 elements, and 7,221,928 elements, respectively (Figure 9). Numerical results were deemed mesh-independent when difference in regional pressure was <1% between two successive meshes.

Regional pressure differences were <1% between the fine and very fine meshes for both Post-S1 and Pre-S2 models. This level of convergence within the pressure field ensures that these models are mesh independent. Furthermore, the high-frequency oscillations within the PA flow field remain present for all levels of refinement for both Post-S1 and Pre-S2 models (Figure 9). This indicates that the high-frequency components are not a result of numerical error but represent a true physical phenomenon. It is worth noting that as the mesh becomes more refined, the high-frequency components become more pronounced in both models.

## Discussion

### Summary

**FIGURE 10:**
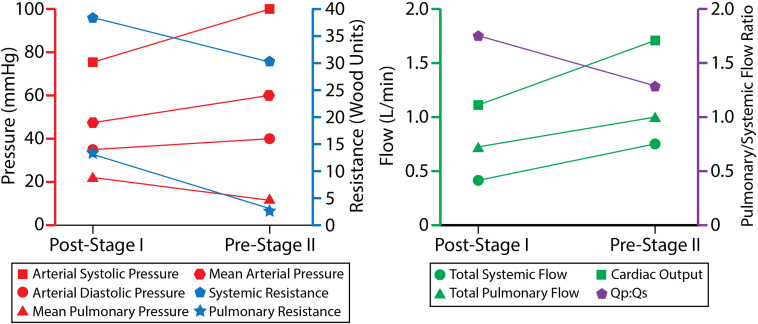
The evolution of hemodynamic indices from ∼7 days of age at post-S1 to ∼5 months of age at pre-S2.

Fluid-structure interaction models of post-S1 and pre-S2 HLHS patients were constructed and calibrated to match *in vivo* hemodynamic and morphological data found in literature. Ultimately, we sought to leverage imaging data, literature data, and computational modeling to enhance our limited understanding of HLHS hemodynamics immediately after stage I palliation. Furthermore, by developing a calibrated pre-S2 model, we were able to describe key hemodynamic changes between the two surgical stages. The evolution of key hemodynamic indices (pressure, flow, resistance, cardiac output, and Qp:Qs ratio) is given in Figure 10.

### Evolution of Hemodynamic Indices From Stage I to Stage II

Pulmonary artery pressure is a key unknown hemodynamic parameter at post-S1 mainly due to the need for invasive cardiac catheterization and the risks involved with introducing a catheter through the systemic-to-PA shunt. This work has given us insight into post-S1 pressure. By matching all available hemodynamic and anatomical data within 10%, and using literature data for typical post-S1 shunt “pinching,” the post-S1 model revealed a PA mean pressure of 22 mmHg (Figure 8). This elevated PA mean pressure is a key finding of this paper and is greater than the pre-S2 mean pressure (14 mmHg). Ideally, we could corroborate this finding with previously published values of post-S1 PA pressure and hemodynamic; however, this data has not been published on up to this point.

The mean systemic and pulmonary resistances decreased from post-S1 to pre-S2: from 37.1 to 31.2 Wood units in systemic resistance, and from 14.8 to 3.8 Wood units in pulmonary resistance. This is in line with what is seen in growing neonates, since distal vascular resistance decreases as vessel and lung maturation ensues (Lucas et al., 1961). This decrease in pulmonary resistance likely drives the increase in mean PA flow at pre-S2 (0.97 L/min, 25% increase from 0.75 L/min at post-S1), despite the larger resistance due to the smaller anastomosis diameter in pre-S2 (2.9 mm) relative to post-S1 (3.15 mm) (see section “Geometric Modeling and Mesh Generation”), which would tend to limit pulmonary flow. The decrease in systemic resistance resulted in a proportionately larger increase in systemic flow and therefore a more balanced Qp:Qs ratio (from 1.8 to 1.3), which nonetheless remained at a sustainable value for lung perfusion at pre-S2.

The compliance of the head and neck vessels (RC, RS, LC, and LS) decreases over time while the compliance of the DAo, LPA, and RPA increases. Broadly, compliance is the change in volume divided by the change in pressure. ΔV is the integral of the flow, which is given by changes in the Qp:Qs ratio. As the body grows, a greater percentage of the cardiac output is directed to the lower body (DAo). Therefore, the ΔV increases, and so does the compliance. As for the LPA and RPA, the ΔP goes down and this also explains why the compliance increases over time. Furthermore, the DAo has more compliance than the LPA and RPA. This can also be understood through the relationship between compliance, changes in volume, and changes in pressure. For the pre-S2 case, in particular, where data is available for ΔP and for the volumes going to DAo and each of the PAs, the calibrated values of distal compliances for the different branches of the model (which allow to match the available population data) do suggest that the total compliance distal to the aorta in the systemic circulation is larger than each of the distal compliances of the PAs. This trend has also been observed in previous computational analyses of HLHS patients (Arthurs et al., 2017).

Finally, despite HLHS patients experiencing volatility within the hours following stage 1 palliation, changes of macroscopic hemodynamic data used to calibrate the simulated models (aortic blood pressure, pulmonary-to-systemic flow ratio, etc.) are often either not statistically significant (Rychik et al., 2000; Pizarro and Norwood, 2003; Cua et al., 2006) or stabilize after day 2 (Mair et al., 2003). When available, data was collected at 48 h post-procedure.

### Disturbed Pulmonary Artery Hemodynamics

The observed elevated PA mean pressure, disturbed flow, and WSS maps suggest that HLHS patients are exposed to suboptimal conditions immediately after surgical reconstruction. The oscillatory hemodynamics are also present at the time of pre-S2 assessment, indicating that these conditions are experienced throughout the interim period between stage I and II (Figure 6). The elevated values of WSS in the PAs are the consequence of the high flows through the shunt necessary to ensure adequate PA flow throughout the pre-S2 period, as the shunt becomes relatively smaller compared to the somatic growth of the patient; this could be a key contributor to complications following stage I palliation.

### Uncertainty of Anastomosis Geometric Definition

Calibrated analyses (i.e., reproducing all available flow data) performed in post-S1 and pre-S2 geometries with perfectly unrestricted shunts (uniform 3.5 mm diameter without “pinching” at the anastomoses), rendered PA mean pressures of 40 and 24 mmHg, respectively. With the calibrated models producing such high pressures, it was determined that we were not capturing some aspect of shunt geometry that resulted in an additional pressure decrease from the systemic to the pulmonary circulation. Thus, it was decided that an alteration in shunt geometry was required to produce such additional pressure drop. Literature studies revealed that immediately following suturing of a PTFE graft to a native vessel, there is often a small degree of pinching at each anastomosis (Dobrin et al., 1998). This pinching becomes even more severe during the interim period between stages I and II as a shunt stenosis begins to develop at each anastomosis (Gladman et al., 1997; Wells et al., 2005).

With PA hemodynamic data available for pre-S2, determining the degree of pinching at the anastomosis constituted an inverse problem. From population-based data, the required pressure decrease across the shunt (60.5–14.3 = 46.3 mmHg, see Table 1) was known, so the degree of pinching was parametrically altered until the desired pressure drop was attained. It was found that a 17% decrease in diameter at each anastomosis was required to reproduce population-based PA hemodynamics. This degree of diameter reduction is in line with what is commonly seen in patients who experience shunt stenosis (Gladman et al., 1997; Wells et al., 2005). Since there is no population-based PA hemodynamic data available for post-S1, the degree of shunt pinching was modeled purely based on the available data on artery-to-PTFE graft anastomoses found in literature (Dobrin et al., 1998). It was found that when suturing a PTFE graft to a native vessel there is a ∼10% decrease in shunt diameter. This diameter reduction was applied to the post-S1 model.

### Limitations

Our study has a few of limitations. First, the starting point of the study was image data corresponding to a central shunt patient, instead of a mBTT patient. Given that the purpose of the image data was to build a model in 3D space that would be subsequently heavily modified to reflect population average dimensions, the quality of the image data (and not the type of shunt) was the most important factor considered when choosing the MRI data. Second, this study only investigated the Norwood procedure with a 3.5 mm mBTTs. Although this surgical configuration and shunt size is widely utilized across many clinical centers; in reality, there are numerous different shunt sizes and surgical configurations (i.e., Sano shunt, central shunt) that could affect the outcome of the data presented. It is our hope that the presented simulations give insight into this specific patient “class,” but also a deeper understanding of what post-operative conditions could look like for HLHS patients undergoing any type of surgical approach. This warrants further investigation and the development of representative models of HLHS patients with other surgical configurations to gain a comprehensive understanding of conditions following the Norwood procedure. Third, average material properties (i.e., vessel stiffness, wall thickness) were applied circumferentially, and therefore did not capture the spatial variation in stiffness in the reconstructed region of the aorta. Ideally, the much stiffer graft material properties would only be applied on the corresponding location of the reconstructed aorta and the remainder would be considered native tissue. Fourth, we’ve shown mesh-independence in both the flow and pressure fields of our models; however, we have not shown mesh-independence for other hemodynamic quantities (i.e., WSS). Since the hemodynamics (and thus the WSS) are substantially disturbed for both post-S1 and pre-S2 cases, the models are likely to be subject to considerably slow convergence rates in the WSS field (Les et al., 2010). In this study, our quantities of interest are flow and pressure, and for those we have demonstrated adequate mesh independence in our results. Finally, the fixed effect model requires a standard deviation for calculating weighted averages. However, some studies within the literature review did not report the standard deviation of measured parameters. In this case, a method commonly applied to approximate standard deviation (or variance) from reported median, range, and sample size was used (Hozo et al., 2005).

## Conclusion

In this study, we combined imaging data, population data, and computational modeling to enhance the current understanding of hemodynamics following the Norwood procedure. Following reconstruction, patients are immediately exposed to suboptimal hemodynamic conditions–elevated PA pressure, oscillatory hemodynamics, and high WSS. Many of these conditions are still present at the time of stage II palliation. In the future, we seek to alter shunt design and configuration to minimize the degree of flow disturbances. We hypothesize that minimization of these flow disturbances would lead to more favorable hemodynamics in the interim period between surgical stages and therefore improve the outcomes of HLHS patients.

## Data Availability Statement

The raw data supporting the conclusions of this article will be made available by the authors, without undue reservation.

## Ethics Statement

The studies involving human participants were reviewed and approved by University of Michigan Medical School Institutional Review Board (IRBMED). Written informed consent from the participants’ legal guardian/next of kin was not required to participate in this study in accordance with the national legislation and the institutional requirements.

## Author Contributions

JP wrote the manuscript, created the figures, performed and analyzed the literature review, built the geometric models, ran the simulations, processed data, and analyzed simulation results. JL acquired clinical data and assisted in manuscript preparation. AS and CF provided analysis of literature review and assisted in manuscript preparation. RG, CF, and AS developed concepts and assisted in manuscript preparation. CF provided analysis of simulation results. All authors contributed to the article and approved the submitted version.

## Conflict of Interest

The authors declare that the research was conducted in the absence of any commercial or financial relationships that could be construed as a potential conflict of interest.

## Footnotes

1 www.crimson.software
